# Discrimination of benign, atypical, and malignant peripheral nerve sheath tumours in neurofibromatosis type 1 – intraindividual comparison of positron emission computed tomography and diffusion-weighted magnetic resonance imaging

**DOI:** 10.1186/s13550-024-01189-0

**Published:** 2024-12-27

**Authors:** Inka Ristow, Ivayla Apostolova, Michael G. Kaul, Maria Stark, Antonia Zapf, Marie-Lena Schmalhofer, Victor F. Mautner, Said Farschtschi, Gerhard Adam, Peter Bannas, Johannes Salamon, Lennart Well

**Affiliations:** 1 Department of Diagnostic and Interventional Radiology and Nuclear Medicine, University Medical Center Hamburg-Eppendorf, Martinistraße 52, 20246 Hamburg, Germany; 2 Institute of Medical Biometry and Epidemiology, University Medical Center Hamburg-Eppendorf, Hamburg, Germany; 3 Department of Neurology, University Medical Center Hamburg-Eppendorf, Hamburg, Germany; 4 Department of Diagnostic and Interventional Radiology, Medical Care Center Beste Trave, Bad Oldesloe, Germany

**Keywords:** ADC, Atypical neurofibroma, DWI, MPNST, MRI, NF1, Nerve sheath tumour, PET, SUV

## Abstract

**Background:**

To intraindividually compare the diagnostic performance of positron emission computed tomography (F-18-FDG-PET/CT) and diffusion-weighted magnetic resonance imaging (DW-MRI) in a non-inferiority design for the discrimination of peripheral nerve sheath tumours as benign (BPNST), atypical (ANF), or malignant (MPNST) in patients with neurofibromatosis type 1 (NF1).

**Results:**

In this prospective single-centre study, thirty-four NF1 patients (18 male; 30 ± 11 years) underwent F-18-FDG-PET/CT and multi-b-value DW-MRI (11 b-values 0 – 800 s/mm²) at 3T. Sixty-six lesions corresponding to 39 BPNST, 11 ANF, and 16 MPNST were evaluated. Two radiologists independently assessed the maximum standardized uptake value (SUV_max_) and mean and minimum apparent diffusion coefficient (ADC_mean/min_) as well as the ADC in areas of lowest signal intensity in each lesion (ADC_dark_). The AUCs of DW-MRI and F-18-FDG-PET/CT were compared to determine whether the ADC is non-inferior to SUV_max_ (non-inferiority margin equal to -10%). Follow-up of ≥ 24 months (BPNST) or histopathological evaluation (MPNST + ANF) served as diagnostic reference standard. Both SUV_max_ and ADC parameters demonstrated good diagnostic accuracy (AUC_SUVmax_ 94.0%; AUC_ADCmean/min/dark_ 91.6% / 90.1% / 92.5%). However, non-inferiority could not be demonstrated for any of the three ADC parameters (lower limits of the confidence intervals of the difference between the AUC of ADC_mean/min/dark_ and SUV_max_ -12.9% / -14.5% / -11.6%). Inter-rater reliability was excellent for both imaging techniques (Krippendorff’s alpha all > 0.94).

**Conclusions:**

Both PET/CT-derived SUV_max_ and MRI-derived ADC allow sensitive and non-invasive differentiation of benign and (pre)-malignant peripheral nerve sheath tumours. Nevertheless, DW-MRI cannot be considered as non-inferior to F-18-FDG-PET/CT in this prospective single-centre study.

**Supplementary Information:**

The online version contains supplementary material available at 10.1186/s13550-024-01189-0.

## Introduction

Neurofibromatosis type 1 (NF1) is an autosomal-dominant neurogenetic disorder with an incidence of about 1:2500–1:3000 [1, 2]. NF1 is caused by a pathogenic variant in the *NF1*-gene on 17q11.2 affecting the tumour-suppressing protein neurofibromin [3]. Patients with NF1 present with a characteristic phenotype including café-au-lait spots, Lisch nodules, skinfold freckling, spinal deformities as well as central and peripheral nerve sheath tumours, with the latter being neurofibromas [4].

Benign peripheral nerve sheath tumours (BPNST), especially internal plexiform neurofibromas originating from the large peripheral nerves, can grow to large size and cause pain and motor dysfunction [5, 6] contributing to reduced quality of life in affected patients [7, 8]. Plexiform neurofibromas require monitoring due to their inherent risk of malignant transformation into highly aggressive sarcomas (MPNST). Early detection of such malignant transformation is critical for individual patient outcome. Malignant transformation of benign tumours is observed in 8 – 16% of NF1 patients and is the most contributing factor to NF1-related death [9, 10].

Neurofibromas with cytological atypia or hypercellularity are considered atypical neurofibromas (ANF). Furthermore, the term atypical neurofibromatous neoplasm of unknown biological potential (ANNUBP) is pathologically defined by the presence of at least two of the following criteria: nuclear atypia, hypercellularity, variable loss of neurofibroma architecture, and/or mitotic activity beyond isolated mitotic figures (> 1/50 high-power field and < 3/10 high-power field) [11]. The terms ANF and ANNUBP are not identical but have been used interchangeably, leading to potential confusion. However, both ANF and ANNUBP are considered pre-malignant tumours with distinct epigenetic profiling different from BPNST and MPNST [12] which are not prone to metastasis or recurrence after complete resection [11, 13].

Magnetic resonance imaging (MRI) with diffusion-weighted imaging is the gold standard imaging technique for long-term monitoring of NF1-associated tumours due to its excellent soft tissue contrast [14]. Further advantages of MRI are that a contrast agent does not necessarily have to be administered and that the patient is not exposed to ionizing radiation. According to current tumour surveillance guidelines for NF1 patients, whole-body MRI should be performed at least at the transition from childhood to adulthood to assess internal tumour burden, which has been correlated with the risk of developing an MPNST [15]. Follow-up scans should therefore be planned under consideration of the respective internal tumour load.

Beyond the evaluation of morphological tumour characteristics, including volumetric assessment of internal tumour burden, the use of diffusion-weighted MRI (DW-MRI) as a functional and quantitative imaging technique has been proven useful in detecting transformation into atypical and/or malignant stages [16–22].

18-F-fluorodeoxyglucose positron emission imaging is recommended in case of suspected malignant transformation [15] but is associated with radiation exposure.

Most of the previous studies investigating the diagnostic performance of DW-MRI and/or PET imaging for the discrimination of NF1-associated peripheral nerve sheath tumours are limited to the comparison of benign vs. malignant tumours, hereby neglecting ANF/ANNUBP in their study design [23–31]. In a meta-analysis, the maximum standardized uptake value (SUV_max_) demonstrated high diagnostic accuracy for the discrimination of benign and malignant peripheral nerve sheath tumours. Highest sensitivity of 0.99 was achieved with an SUV_max_ cut-off value of ≥ 3.5 with a specificity of 0.75 [32]. Regarding DW-MRI, in particular, the mean and minimum apparent diffusion coefficient (ADC_mean/min_), as well as the ADC in the slice with the lowest signal intensity (ADC_dark_) were proposed as quantitative imaging parameters, demonstrating good performance for the discrimination of benign vs. malignant when using an ADC_min_ cut-off value of ≤ 1 × 10^− 3^ mm^2^/s [17, 21] and ≤ 1.54 × 10^− 3^ mm^2^/s for ADC_dark_ [16].

Few studies included atypical tumours in their evaluations by showing that pre-malignant ANF cluster in between the benign and malignant tumours with respect to metabolic activity in 18-FDG-PET imaging as well as diffusion restriction in DW-MRI. For example, Fertitta et al. reported a mean SUV_max_ of 8.3 ± 4.7 for MPNSTs, while SUV_max_ was 5.3 ± 0.6 in neurofibromas with atypia, and 5.6 ± 1.3 in ANNUBP [33]. The observations were consistent with those of Warbey et al. in which the SUV_max_ of atypical neurofibromas clustered between those of benign neurofibromas and MPNST [34]. Adding to this, Ristow et al. provided evidence for an intermediate diffusion restriction profile in ANFs between that of benign and malignant peripheral nerve sheath tumours [22].

However, it should be taken into account that the use of different machines, imaging protocols, image reconstruction techniques, and post-processing software causes variability in F-18-FDG-PET/CT or DW-MRI measurements [35–37]. Therefore, it is not possible to define appropriate diagnostic cut-off values that can be applied with the same degree of reliability in different facilities.

At present, it is not sufficiently clear to what extent diffusion-weighted imaging can be regarded as an equivalent method to discriminate NF1-associated tumours with suspected malignant transformation in routine clinical practice. Data on intraindividual comparison of PET/CT and DW-MRI are missing. Therefore, the purpose of this study was to intraindividually compare the diagnostic performance of positron emission computed tomography (F-18-FDG-PET/CT) and diffusion-weighted magnetic resonance imaging (DW-MRI) for the discrimination of peripheral nerve sheath tumours in patients with neurofibromatosis type 1 (NF1) as benign, atypical, or malignant.

## Materials and methods

This prospective single-centre study was approved by the local ethics committee (Ärztekammer Hamburg; PV4691). Written informed consent to publish was obtained from all patients. All procedures complied with the local data protection guidelines.

### Study population

Inclusion criteria were a confirmed NF1 diagnosis by genetic testing and availability of diffusion-weighted magnetic resonance imaging (DW-MRI) at 3T with multiple b-values as well as F-18-FDG-PET/CT. DW-MRI and matching F-18-FDG-PET/CT had to be no more than six weeks apart. Consecutive recruitment was performed from 08/2014 to 12/2021. Data on the reasons for the imaging, especially the patients’ clinical symptoms such as pain, were not systematically collected. Following the STARD recommendations for reporting diagnostic studies, a flow chart illustrating the participants’ flow through the study is attached to Supplementary Fig. 1. Histopathological evaluation after surgical resection served as the diagnostic reference standard for MPNST and ANF. Resected MPNST were classified according to the grading system of the Fédération Nationale des Centres de Lutte Contre le Cancer (FNCLCC) [38].

As performed previously, tumours were considered benign when no changes in size or appearance were present in follow-up examinations within 24 months [22, 39].

### Imaging

#### DW-MRI data acquisition

MR imaging was performed at 3T (Philips Ingenia, Best, The Netherlands). The detailed scanning protocol was reported previously [16, 22].

In short, local DWI stacks were scanned covering each tumour of interest. The DW-MRI protocol used an axial respiratory-triggered spin-echo planar imaging (EPI) sequence (parallel acquisition; sense factor 2.8; TR 2300 ms; TE 67 ms; echo train length 43; flip angle 90°; slice thickness 3 mm; intersection gap 0 mm; matrix 124 × 122; FOV 270 × 270 mm; voxel size 1.5 × 1.5 × 3 mm; SPAIR; two averages) with eleven b-values (0, 10, 20, 30, 50, 70, 100, 300, 400, 600, 800 s/mm^2^). Scan time of the DW-MRI was about 6 – 7 min.

#### F-18-FDG-PET/CT data acquisition and image reconstruction

The PET imaging protocol required a fasting period of at least 6 h and a blood glucose level of ≤ 200 mg/dl before F-18-FDG administration. The average F-18-FDG injection dose was 287 ± 56 MBq (range 147 – 397 MBq). During the uptake period of 79 ± 18 min (range 44 – 114 min), patients were hydrated orally with water.

From 08/2014 to 11/2018 F-18-FDG-PET/CT imaging was performed using a Gemini GXL 10 scanner PET/CT (Philips Healthcare, Best, The Netherlands; n_tumours_ = 37) and from 12/2018 to 12/2021 using a Vereos whole-body TOF-PET/CT (Philips Healthcare, Best, The Netherlands; n_tumours_ = 29). The detailed imaging protocols for both scanners were reported previously [25, 26, 40, 41].

In brief, PET/CT acquisition was performed from skull vertex to toes in each patient.

Philips Gemini GXL 10 attenuation correction was performed using auxiliary CT with 50 mAs, 120 kV, rotation time 0.5 s, spiral pitch factor 1.1 reconstructed with 5 mm slice thickness (512 × 512 matrix; 1.17 × 1.17 mm pixel size). PET images were acquired with 90 s per bed position for the trunk (60 s for the extremities) and reconstructed using iterative 3DLOR reconstruction algorithm with default parameter settings (e.g., two iterations; no subsets; voxel size 4.0 × 4.0 × 4.0 mm).

PET images acquired with Vereos whole-body TOF-PET/CT were performed with 90 s per bed position for the trunk (60 s extremities) and reconstructed with auxiliary CT-based attenuation and scatter correction using the blob-ordered subset time-of-flight reconstruction algorithm of the system (2 iterations; 10 subsets; reconstructed voxel size 2.0 × 2.0 × 2.0 mm). The attenuation map was generated from the whole-body low-dose CT scan (50 mAs/120 kV; rotation time 0.5; spiral pitch factor 0.8) reconstructed with a slice thickness of 3 mm (matrix size 512 × 512; pixel size 1.17 × 1.17 mm).

### Data analysis

#### DW-MRI data analysis

DW-MRI data were processed as described previously [16, 22] using a self-developed image-analysis framework (qMapIt) [42] extending the open-source software ImageJ [43]. In brief, quantitative parametric maps were calculated by nonlinear regression with pixel-wise fitting of signal intensities over the spectrum of b-values to the corresponding model. A monoexponential function was applied for ADC determination in ImageJ. Regarding ADC measurements, regions of interest (ROIs) were independently placed by the two readers by manual contouring of the tumours in the diffusion images (b-value 50 s/mm^2^). ROIs were placed along tumour margins in three adjacent slices with the largest axial tumour diameter. Averaged values from the three ROIs were calculated to assess the mean and minimum apparent diffusion coefficient (ADC_mean/min_). Additionally, a single ROI was placed along tumour margins at the visually lowest signal intensity to determine ADC_dark_.

#### F-18-FDG-PET/CT data analysis

F-18-FDG-PET/CT data were analyzed using the software ROVER v3.0.61 h (ABX advanced biochemical compounds GmbH, Radeberg, Germany). Data analysis was performed independently by two readers (IR and LW) as described by others [44]. In brief, 3d spheres covering the entire tumour volume were manually placed based on the attenuation-corrected emission data using the non-enhanced CT images for anatomical orientation. The metabolically active part of the peripheral nerve sheath tumour was delineated by an automatic algorithm based on adaptive thresholding. A detailed description of the algorithm has been published elsewhere [45, 46]. When necessary, a click-based manual correction of the ROI was performed to exclude metabolically active adjacent structures, e.g. the brain.

#### Statistical analysis

This article is based on preliminary work aiming to define ADC-based diagnostic cut-off values to classify NF1-associated peripheral nerve sheath tumours as benign, atypical, or malignant [22]. All patients from the former study with DW-MRI and a time-matched F-18-FDG-PET/CT were included in the cohort of the current study.

Considering the preceding study, this article is based on secondary evaluations; the results are therefore presented descriptively. Statistical analysis was performed using the software R, version 4.1.3 [47]. Mean and minimum ADC values from each of the three ROIs per lesion were averaged to ADC_mean_ and ADC_min_. There were no missing values in the complete data set. ANF and ANNUBP were considered together as the ANF group. To compare each ADC and SUV_max_ value between the three tumour groups (BPNST, ANF and MPNST), a linear mixed model was calculated [48] with an ADC or SUV_max_ value as one of the dependent variables, respectively. The tumour group and reader were included as fixed effects and a random-intercept was included for each patient. Estimated marginal means and their pairwise contrasts with two-sided 95% confidence intervals (95%-CI) were calculated. Krippendorff’s alpha with bootstrapped 95%-CI was assessed to quantify interrater agreement [49]. A non-parametric multi-factorial approach was applied to calculate the area-under-the-curve (AUC) of the ADC values and the SUV_max_ value [50]. For this purpose and according to Ristow et al. (2024) [22], the three-level reference standard was dichotomized in two ways of discrimination:

- Discrimination 1: BPNST vs. ANF + MPNST.
- Discrimination 2: BPNST + ANF vs. MPNST.

Considering the clinical implications, the resulting AUC curves of this article are based on Discrimination 1 as the primary comparison, as both suspected ANF or MPNST require further diagnostic workup such as additional F-18-FDG-PET/CT and/or invasive approaches such as biopsy or surgical resection.

To evaluate whether ADC values are non-inferior compared to the SUV_max_, the difference between the ADC value and SUV_max_ with two-sided 95%-CI were calculated, respectively [50, 51]. After careful consideration, taking into account the specific cohort and clinical question, and aiming to balance realistic discriminatory power and tolerance, the non-inferiority margin was set to -10%, which is within the range of commonly applied non-inferiority margin sizes [52].

To report correlations between the ADC values and SUV_max_, geometric means of two multilevel model coefficients were calculated with two-sided 95%-CI, respectively. The multilevel models contain SUV_max_ and the ADC value as the dependent variable and fixed effect and vice versa. Furthermore, they include the reader as fixed effect and a random intercept per patient.

## Results

### Study population and reference standard

Thirty-four NF1 patients (18 male; mean age 30 ± 11 years, range 12 – 54 years) were identified, corresponding to 39 BPNST, 11 ANF, and 16 MPNST. Fifteen patients had more than one lesion. Among these, two patients had two ANF. No patient had multiple MPNST. There was no BPNST that underwent subsequent transition into an MPNST. All ANF were excised, therefore none transformed into an MPNST.

Histopathological reports following MPNST resection revealed 1 grade I, 6 grade II, and 9 grade III tumours according to Fédération Nationale des Centres de Lutte Contre le Cancer (FNCLCC).

### Distribution of ADC parameters and SUVmax of BPNSTs, ANFs, and MPNSTs

**Fig. 1:**
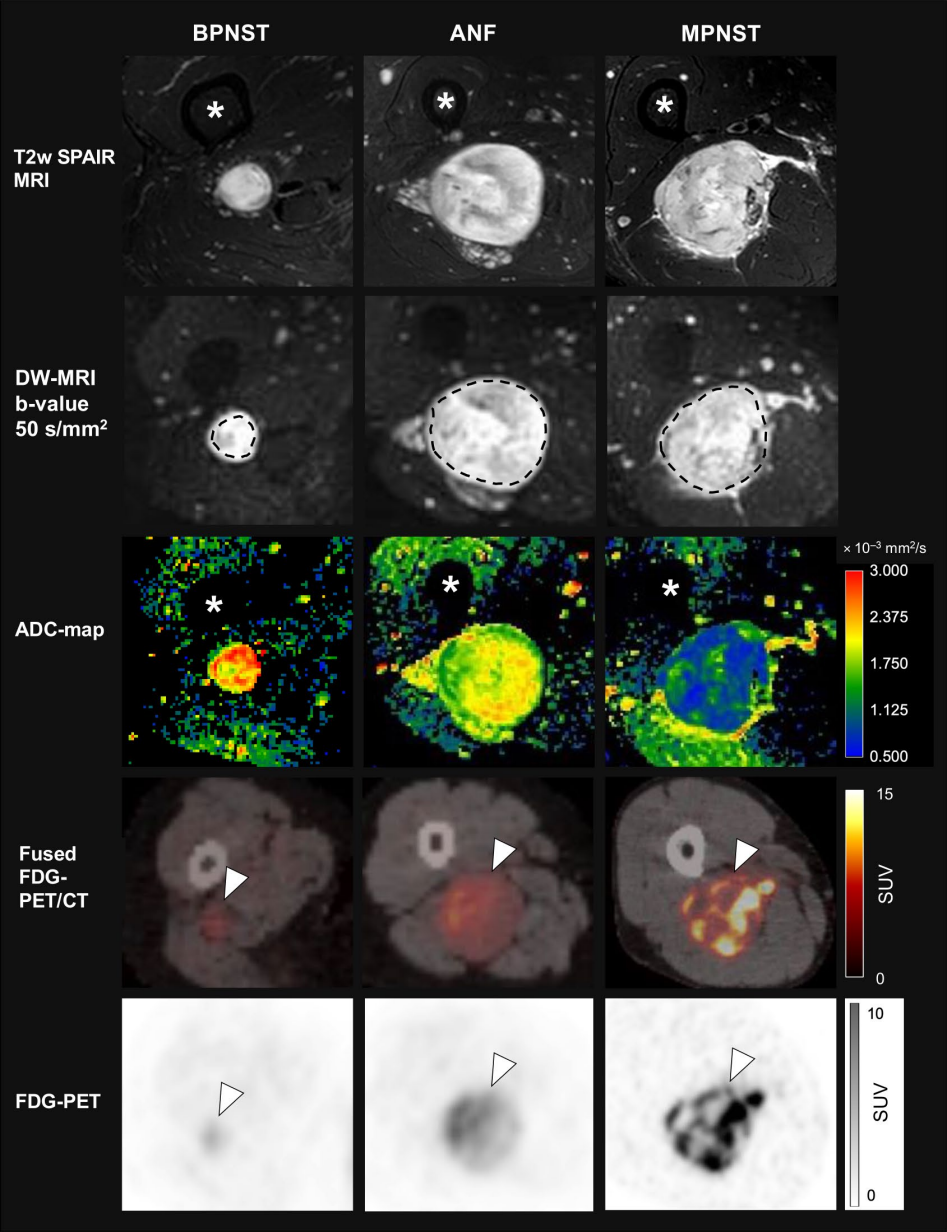
Comparison of T2-weighted SPAIR MRI, parametric ADC maps, and F-18-FDG-PET/CT in three different NF1 patients with nerve sheath tumours of the thigh. Left column: BPNST (arrowhead) in a 54-year-old female; middle column: ANF (arrowhead) in a 49-year-old female, right column: MPNST (arrowhead) in a 31-year-old male. First row: transverse slices of a T2-weighted SPAIR sequence at 3T (* depict the femoral bone); second row: ROI contouring along the tumour margins in diffusion images (b-value 50 s/mm^2^); third row: corresponding parametric ADC maps; fourth row: fused F-18-FDG-PET/CT; fifth row: F-18-FDG-PET. BPNST = benign peripheral nerve sheath tumour; ANF = atypical neurofibroma; MPNST = malignant peripheral nerve sheath tumour; SUV = standardized uptake value

**Fig. 2:**
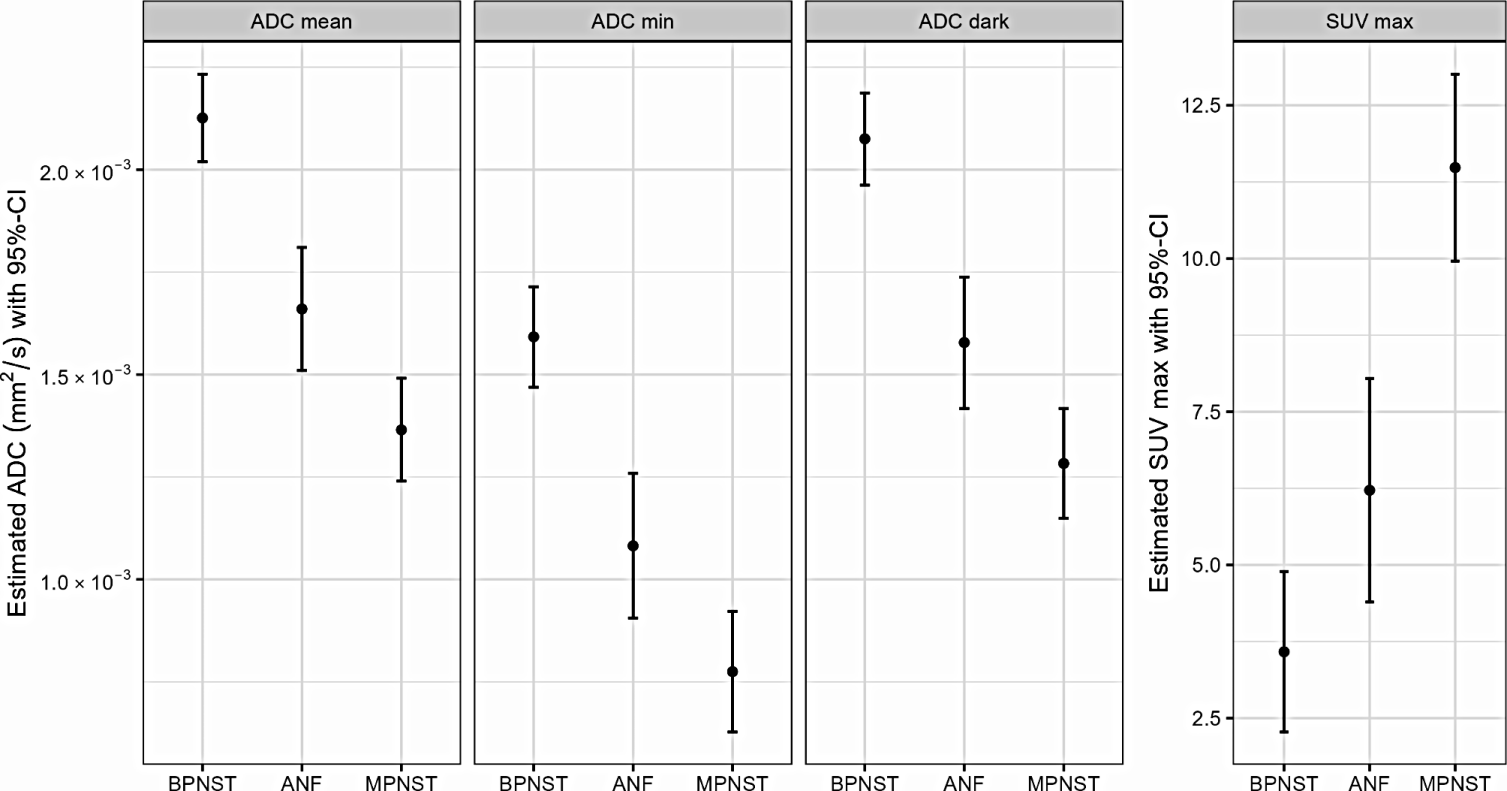
Estimated marginal means for ADC_mean_, ADC_min_, ADC_dark_, and SUV_max_ of benign, atypical, and malignant peripheral nerve sheath tumours. ADC_mean_ = mean apparent diffusion coefficient; ADC_min_ = minimum apparent diffusion coefficient; ADC_dark_ = apparent diffusion coefficient in the slice with lowest signal intensity; BPNST = benign peripheral nerve sheath tumour; ANF = atypical neurofibroma; MPNST = malignant peripheral nerve sheath tumour; CI = confidence interval

Figure 1 shows exemplary axial T2w images and ADC maps of a BPNST, ANF, and MPNST in the thigh of three different patients, including the respective ROI placement and the corresponding F-18-FDG-PET/CT. Estimated marginal means and 95%-CI for the three evaluated DW-MRI-derived ADC parameters and F-18-FDG-PET/CT-derived SUV_max_ are given in Table 1A and are visualized in Fig. 2. Results of mixed models used to estimate the marginal means for comparing ADC values between tumour groups are given in Supplementary Table 1.

Differences of the ADC values and p-values of the pairwise comparisons are displayed in Table 1B. All pairwise comparisons of BPNST vs. ANF vs. MPNST group revealed p-values < 0.01.

### ROC-AUC analysis for BPNST, ANF, and MPNST discrimination

**Fig. 3:**
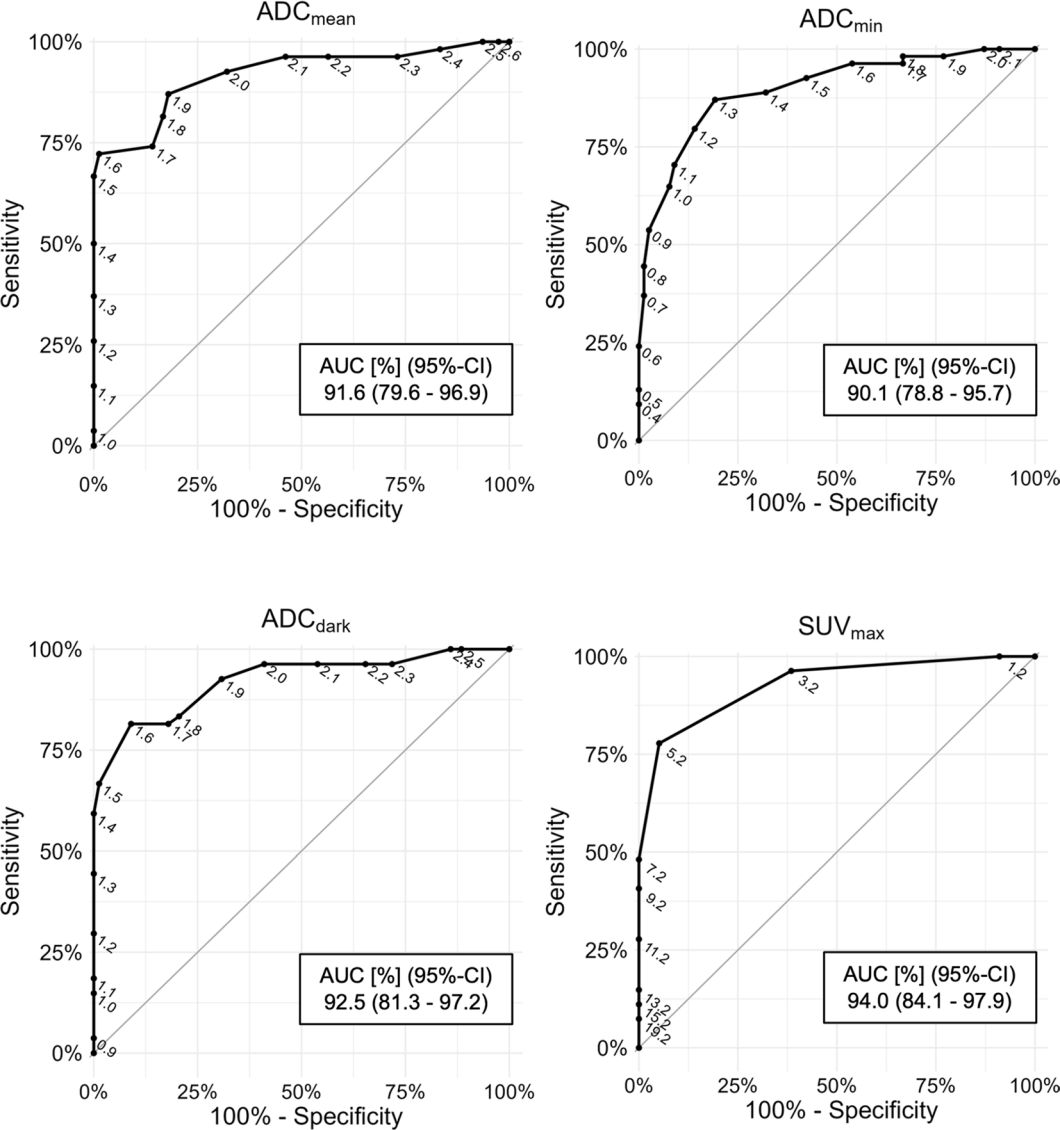
ROC AUC curves visualizing diagnostic performance for the primary comparison BPNST vs. ANF + MPNST (Discrimination 1). Top left: mean apparent diffusion coefficient (ADC_mean_); top right: minimum apparent diffusion coefficient (ADC_min_); mean apparent diffusion coefficient (ADC_mean_); bottom left: apparent diffusion coefficient in the slice with the lowest signal intensity (ADC_dark_); bottom right: maximum standardized uptake value (SUV_max_). Both DW-MRI and F-18-FDG-PET/CT reveal good diagnostic performance to discriminate BPNST from ANF or MPNST with mean AUCs > 90%

Figure 3 visualizes the ROC curves of Discrimination 1 (BPNST vs. ANF + MPNST) for the three ADC parameters and SUV_max_. Corresponding AUC values including 95%-CI for the four parameters are shown in Table 2 for Discrimination 1 and 2, respectively. Regarding Discrimination 1, the AUC was 91.6% (95%-CI: 79.6 – 96.9) for ADC_mean_, 90.1% (95%-CI: 78.8 – 95.7) for ADC_min_, 92.5% (81.3 – 97.2) for ADC_dark_, and 94.0% (84.1 – 97.9) for SUV_max_. Potential ADC-based diagnostic cut-off values for BPNST, ANF, and MPNST discrimination have already been reported in previous work [21]. Potential SUV_max_-based diagnostic cut-off values for BPNST, ANF, and MPNST discrimination are provided in Supplementary Table 2.

**Table 1:** Estimated marginal means and pairwise comparisons of ADC_mean_, ADC_min_, ADC_dark_, and SUV_max_

A) Estimated marginal means	ADC parameter		BPNST (*n* = 78*)	ANF (*n* = 22*)	MPNST (*n* = 32*)	
	ADC_mean_ [× 10^− 3^ mm^2^/s] (95%-CI)		2.13 (2.02 – 2.23)	1.66 (1.51 – 1.81)	1.37 (1.24 – 1.49)	
	ADC_min_ [× 10^− 3^ mm^2^/s] (95%-CI)		1.59 (1.47 – 1.71)	1.08 (0.91 – 1.26)	0.78 (0.63 – 0.92)	
	ADC_dark_ [× 10^− 3^ mm^2^/s] (95%-CI)		2.08 (1.96 – 2.19)	1.58 (1.42 – 1.74)	1.28 (1.15 – 1.42)	
	SUV_max_ (95%-CI)		3.58 (2.28 – 4.89)	6.22 (4.40 – 8.04)	11.48 (9.96 – 13.01)	
**B) Pairwise comparisons**	**ADC parameter**		**BPNST vs. ANF**	**BPNST vs. MPNST**	**ANF vs. MPNST**	
	ADC_mean_	Difference [× 10^− 3^ mm^2^/s] (95%-CI)	0.47 (0.30 – 0.63)	0.76 (0.63 – 0.89)	0.30 (0.12 – 0.47)	
		p-value	< 0.0001	< 0.0001	0.0011	
	ADC_min_	Difference [× 10^− 3^ mm^2^/s] (95%-CI)	0.51 (0.31 – 0.70)	0.82 (0.66 – 0.97)	0.31 (0.10 – 0.52)	
		p-value	< 0.0001	< 0.0001	0.0046	
	ADC_dark_	Difference [× 10^− 3^ mm^2^/s] (95%-CI)	0.50 (0.32 – 0.67)	0.79 (0.65 – 0.93)	0.30 (0.11 – 0.49)	
		p-value	< 0.0001	< 0.0001	0.0027	
	SUV_max_	Difference [× 10^− 3^ mm^2^/s] (95%-CI)	-2.64 (-4.60 – -0.67)	-7.90 (-9.43 – -6.38)		-5.27 (-7.37 – -3.16)
		p-value	0.0091	< 0.0001		< 0.0001

ADC_mean_ = mean apparent diffusion coefficient; ADC_min_ = minimum apparent diffusion coefficient; ADC_dark_ = apparent diffusion coefficient in the slice with lowest signal intensity; BPNST = benign peripheral nerve sheath tumour; ANF = atypical neurofibroma; MPNST = malignant peripheral nerve sheath tumour; CI = confidence interval. * N was assessed by multiplication of the number of tumours (39 BPNSTs, 11 ANFs, 16 MPNSTs) by the number of readers (*n* = 2)

**Table 2:** ROC-analysis-derived AUC for DW-MRI-based ADC parameters and F-18-FDG-PET/CT-based SUV_max_

Comparison	AUC ADC_mean_ [%] (95%-CI)	AUC ADC_min_ [%] (95%-CI)	AUC ADC_dark_ [%] (95%-CI)	AUC SUV_max_ [%] (95%-CI)
BPNST vs. ANF + MPNST	91.6 (79.6 – 96.9)	90.1 (78.8 – 95.7)	92.5 (81.3 – 97.2)	94.0 (84.1 – 97.9)
BPNST + ANF vs. MPNST	95.8 (87.7 – 98.7)	93.3 (86.3 – 96.9)	94.4 (86.1 – 97.9)	91.5 (73.9 – 97.6)

ADC_mean_ = mean apparent diffusion coefficient; ADC_min_ = minimum apparent diffusion coefficient; ADC_dark_ = apparent diffusion coefficient in the slice with lowest signal intensity; SUVmax = maximum standardized uptake value; BPNST = benign peripheral nerve sheath tumour; ANF = atypical neurofibroma; MPNST = malignant peripheral nerve sheath tumour; CI = confidence interval

Best SUV_max_-based BPNST vs. ANF + MPNST discrimination was obtained using a cut-off value of 5 resulting in a sensitivity of 85.2% (62.7 – 95.2) and specificity of 92.3% (77.4 – 97.7).

### Inter-rater reliability (Krippendorff’s alpha)

The agreement between the two readers was excellent for all investigated parameters. Krippendorff’s alpha for ADC_mean_ was 0.98 (95%-CI: 0.95–0.99). For ADC_min_, Krippendorff’s alpha was 0.94 (0.85 – 0.98), for ADC_dark_ 0.97 (0.93 – 0.99), and for SUV_max_ it was > 0.99 (0.92 – 1.00).

### Test for non-inferiority of DW-MRI compared to F-18-FDG-PET/CT

**Fig. 4:**
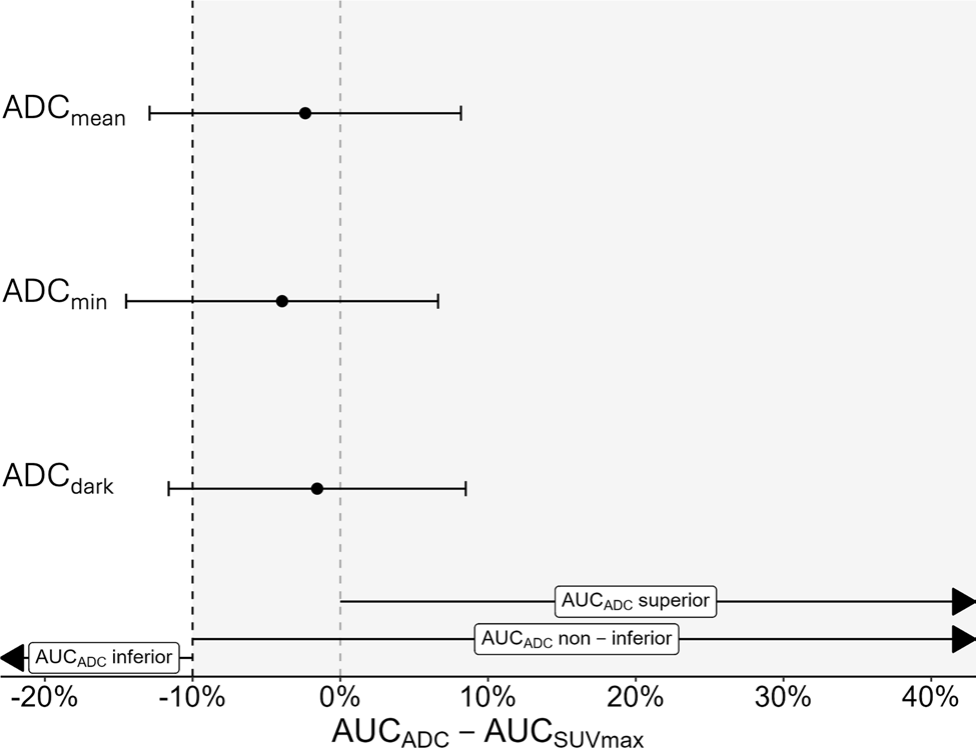
Non-inferiority plot of the three DW-MRI-derived parameters for the primary comparison BPNST vs. ANF + MPNST. In all three evaluated DW-MRI-based parameters, the lower confidence interval of the differences of the AUCs of the ADC and SUV_max_ was below the a priori-defined non-inferiority limit of -10%. Consequently, non-inferiority could not be demonstrated

A non-inferiority plot of the three DW-MRI-derived parameters compared to SUV_max_ for Discrimination 1 (BPNST vs. ANF + MPNST) is visualized in Fig. 4. The difference between the AUC of ADC_mean_ and SUV_max_ was -2.4% (-12.9% – 8.17%). Regarding ADC_min_ and SUV_max_, the difference between the AUCs was -3.9% (-14.5% – 6.6%), and for ADC_dark_ the difference was -1.6% (-11.6% – 8.5%). Non-inferiority could hence not be demonstrated for any of the three ADC parameters, as the lower limits of the confidence intervals (-12.9% / -14.5% / -11.6%) were below the a priori-defined non-inferiority margin of -10%.

### Correlation between ADC and SUVmax

**Fig. 5:**
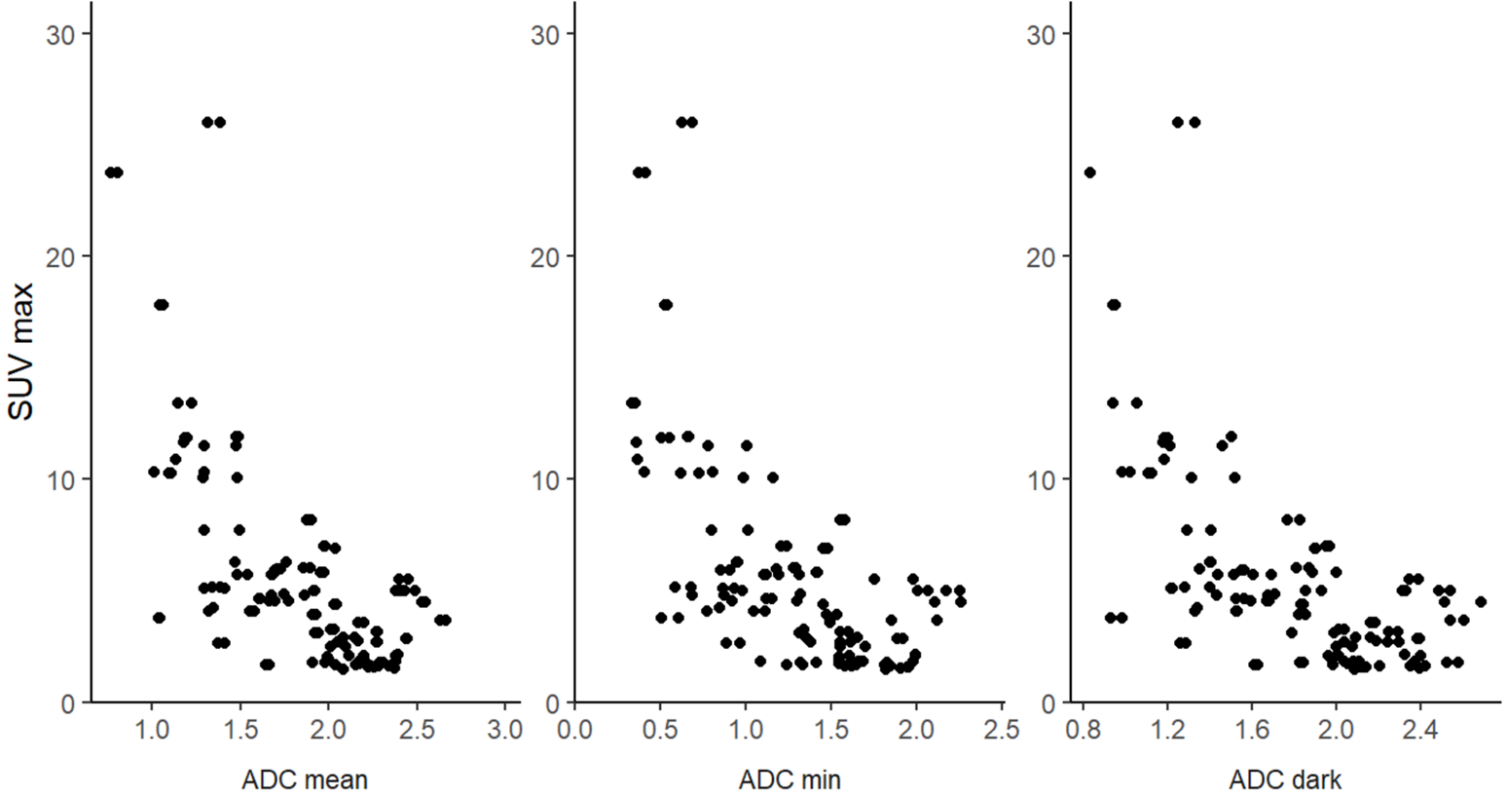
Correlation plots of SUV_max_ and the three DW-MRI-derived ADC parameters for the primary comparison BPNST vs. ANF + MPNST. Correlation coefficients revealed moderate negative association for ADC_mean_ (*r* = -0.60), ADC_min_ (*r* = -0.58), and ADC_dark_ (*r* = -0.58) with SUV_max_

There was a negative correlation between the three ADC parameters and SUV_max_ for comparison 1 (BPNST vs. ANF + MPNST). Correlation coefficients revealed moderate negative association for ADC_mean_ (*r* = -0.60 (95%-CI: -0.74 – -0.46)), ADC_min_ (*r* = -0.58 (95%-CI: -0.72 – -0.44)), and ADC_dark_ (*r* = -0.58 (95%-CI: -0.72 – -0.44)) with SUV_max_, respectively (Fig. 5).

## Discussion

In this study, we intraindividually compared the diagnostic accuracy of F-18-FDG-PET/CT and DW-MRI for the discrimination of NF1-associated peripheral nerve sheath tumours as benign, atypical, or malignant. A non-inferiority study design with a margin of -10% was conducted to compare the AUC of F-18-FDG-PET/CT-derived maximum standardized uptake value (SUV_max_) with the AUC of DW-MRI-derived mean and minimum apparent diffusion coefficient (ADC_mean/min_) as well as the ADC in the slice with the lowest signal intensity (ADC_dark_). Both imaging techniques demonstrated utility with good overall diagnostic accuracy (AUC_SUVmax_ 94.0%; AUC_ADCmean/min/dark_ 91.6% / 90.1% / 92.5%) and excellent inter-rater reliability (Krippendorff’s alpha all ≥ 0.94).

However, non-inferiority could not be demonstrated for any of the three ADC parameters (lower limits of the confidence intervals of the difference between the AUC of AUC_ADCmean/min/dark_ and SUV_max_ -12.9% / -14.5% / -11.6%). We therefore conclude that despite the advantages of ionization radiation-free DW-MRI, PET imaging should still be considered the diagnostic gold standard in the case of suspected malignant transformation in peripheral nerve sheath tumours. Nevertheless, MRI remains an important mainstay in the diagnosis of NF1-associated peripheral nerve sheath tumours, particularly given its better suitability for screening purposes. In contrast, the collective is pre-selected in PET/CT, as there is already a suspicion of MPNST.

In line with previous work and according to their histopathological classification as pre-malignant tumours, the SUV_max_ as well as the ADC of the ANF clustered between the BPNST and MPNST [22, 33, 34].

It should be noted that the more complex statistical approach now involving three tumour groups (BPNST, ANF, and MPNST) instead of only two in most of the previous literature (BPNST vs. MPNST) is reflected by a lower discriminatory power. For example, a meta-analysis by Martin et al. reported pooled diagnostic accuracies for differentiating between BPNST and MPNST were higher for SUV_max_, with the best accuracy being achieved with a cut-off value of 3.5 (sensitivity 0.99, specificity 0.75) [32]. In accordance, the best accuracy was achieved in our data for the BPNST vs. ANF + MPNST comparison with a comparable cut-off value of 4.0 (sensitivity 0.93, specificity 0.72; Supplementary Table 2).

It should be taken into account that besides the SUV_max_, which is quickly measurable and certainly considered the most widely used semi-quantitative parameter across centres in routine clinical practice, there are also other PET-derived parameters in the literature, which have been evaluated for the differentiation of peripheral nerve sheath tumours in NF1, e.g., the mean SUV, heterogeneity index, or tumour-to-liver, -muscle, and -fat ratio [23–25, 35, 53–55]. We have refrained from including these parameters in the current analysis, as they play a rather secondary role in the current standard of care.

The negative correlation between the F-18-FDG-PET/CT-derived SUV_max_ and the DW-MRI-derived ADC values in our study was comparable to that reported by others for other tumour entities [56–58]. As both high SUV_max_ and low ADC values are associated with tumour malignancy, the identified correlation appears reasonable.

In addition to the use of histomorphological features only, CDKN2A/B mutation analysis is now routinely performed for diagnosing malignant transformation in peripheral nerve sheath tumours. Homozygous loss of this cell cycle regulator is considered an additional marker for the conversion of benign into pre-malignant or malignant peripheral nerve sheath tumours [13, 59, 60]. A review of the records revealed a homozygous loss in only five ANF in our collective, a heterozygous loss in one lesion, and no mutation in the CDKN2A/B gene in 3 ANF. According to the patient chart, the remaining tumours did not undergo any further genetic analyses. Beyond that, other markers - like methylation profiling - seem to strengthen the validity of histopathological analysis [12].

The study has the following limitations: First, the relatively small number of MPNST and ANF as well as the long data acquisition period including two different PET/CT scanners, both of which are explicable by the rarity of the disease. However, this resulted in acquisition of PET images with inherently different spatial resolution, which impacts quantitative assessments including SUV_max_ estimates. Nevertheless, we did not stratify the analysis according to the PET scanners due to the limited sample size. This might have caused a bias to the disadvantage of F-18-FDG-PET/CT resulting in underestimation of its discriminative power. Similarly and despite excellent interrater reliability, the ADC-based cut-off values obtained in our study should not be used at other institutions without previous correlation with institution specific ADC values due to intersystem and intervendor variability of ADC measurements [35, 36].

Second, given the long acquisition period, it should be taken into account that a WHO revision was carried out in 2021 including improved consensus criteria for the histomorphological diagnosis of ANF (referred to in the WHO as ANF or ANNUBP) [61]. As a result, a corresponding variability in tumour classification of ANF vs. ANNUBP cannot be fully ruled out. However, none of the included ANF/ANNUBP was retrospectively assigned the diagnosis MPNST, and ANF and ANNUBP were considered together in the statistical analysis.

Third, the two techniques (DW-)MRI and PET imaging were considered as two separate imaging techniques in this study. Considering the increasing availability of PET/MRI, which allows to correlate information about local tumour metabolism with excellent morphological tumour visualization within one session, future studies must show whether synergistic effects can be achieved to increase diagnostic accuracy to discriminate between benign, atypical, and malignant peripheral nerve sheath tumours.

## Conclusion

In conclusion, both F-18-FDG-PET/CT-derived SUV_max_ and DW-MRI-derived ADC allow sensitive and non-invasive differentiation of benign and (pre)-malignant peripheral nerve sheath tumours in need of further diagnostic workup, e.g., by biopsy. Nevertheless, diffusion-weighted MRI cannot be considered an equivalent method to F-18-FDG-PET/CT in this single-centre study, and more imaging surrogates are desperately needed to provide a sufficient risk stratification for the decision-making process before biopsy and/or surgery.

## Electronic supplementary material

Below is the link to the electronic supplementary material.

Supplementary Material 1

## Data Availability

The datasets generated during and/or analysed during the current study are available from the corresponding author on reasonable request.
