# Supplementary material for: Discrimination of benign, atypical, and malignant peripheral nerve sheath tumours in neurofibromatosis type 1 – intraindividual comparison of positron emission computed tomography and diffusion-weighted magnetic resonance imaging
Source: EJNMMI Res. 2024 Dec 27;14:127. doi: 10.1186/s13550-024-01189-0 (PMC11680535; doi:10.1186/s13550-024-01189-0)
Supplement: Supplementary file 1 — Supplementary Material 1 [file 13550_2024_1189_MOESM1_ESM.docx]

**Supplement**

***Supplementary Fig. 1) Flow chart to illustrate in- and exclusion of participants into the final study population.***

**
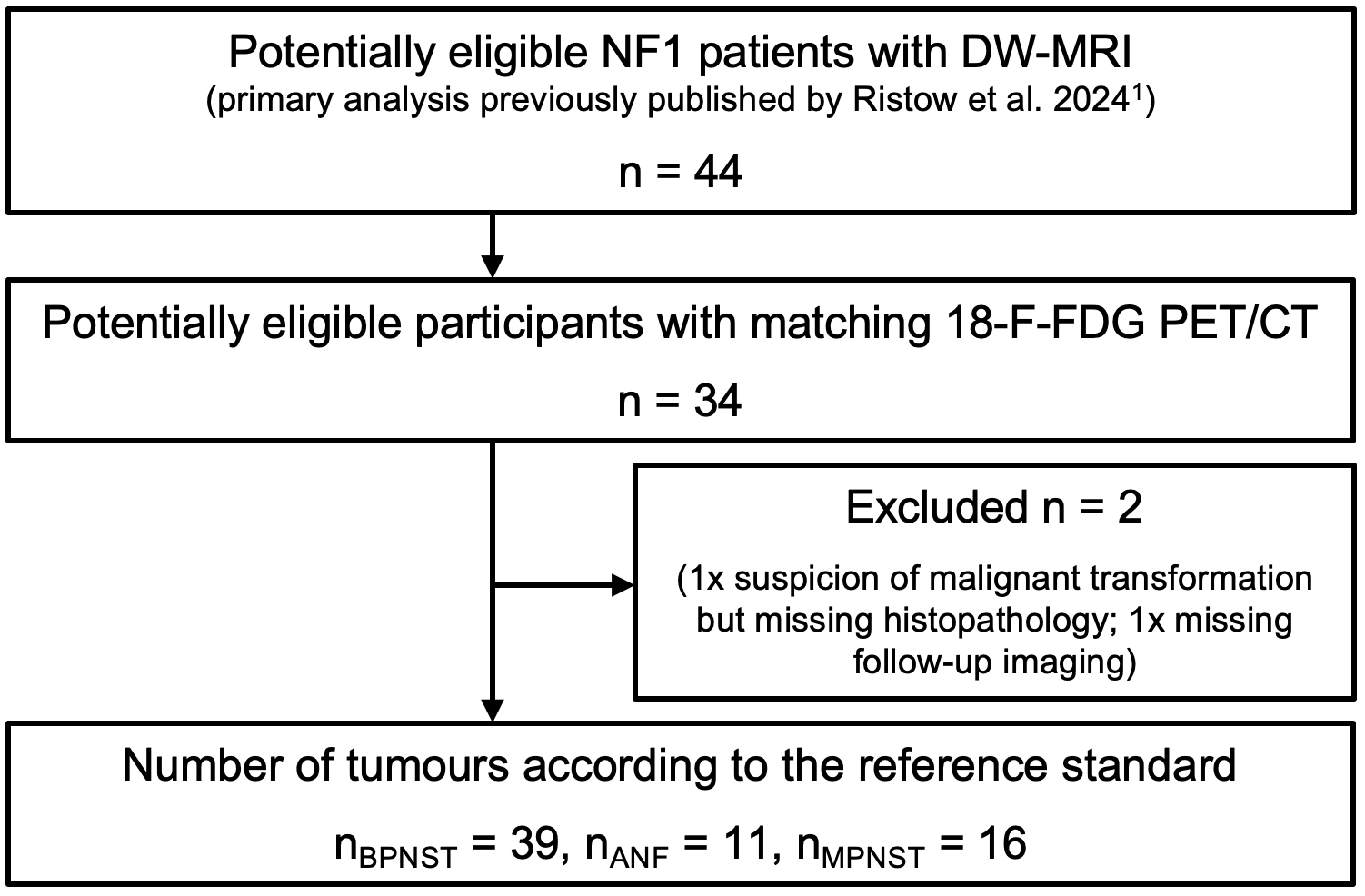
**

^1^ Ristow I, Kaul MG, Stark M, Zapf A, Riedel C, Lenz A, Mautner VF, Farschtschi S, Apostolova I, Adam G, Bannas P, Salamon J, Well L. Discrimination of benign, atypical, and malignant peripheral nerve sheath tumors in neurofibromatosis type 1 using diffusion-weighted MRI. Neurooncol Adv. 2024. 6(1):vdae021. doi: 10.1093/noajnl/vdae021.

***Supplementary Tab. 1) Estimates of regression coefficients with 95% confidence intervals and p-values of mixed models to compare ADC values and SUV_max_ between tumor groups. A random-intercept for each patient is included. Tumor group and reader are included as fixed effects. Unexplained residual variance within patients is denoted by*** $\boldsymbol{\sigma}^{\boldsymbol{2}}$***, variance between patients is denoted by*** $\boldsymbol{\tau}_{\boldsymbol{00}}$ ***and number of patients is given with N.***

|  | | ADC_mean_ | | | | | | | | | | ADC_min_ | | | | | | | | | | | | ADC_dark_ | | | | | | | | | | | | SUV_max_ | | | | | | | | | | | | | |
| --- | --- | --- | --- | --- | --- | --- | --- | --- | --- | --- | --- | --- | --- | --- | --- | --- | --- | --- | --- | --- | --- | --- | --- | --- | --- | --- | --- | --- | --- | --- | --- | --- | --- | --- | --- | --- | --- | --- | --- | --- | --- | --- | --- | --- | --- | --- | --- | --- | --- |
| *Predictors* | *Esti-mates* | | | | *CI* | | | | *p-value* | | | *Esti-mates* | | | *CI* | | | | *p-value* | | | | *Esti-*  *mates* | | | | *CI* | | | | *p-value* | | | | | | *Esti-*  *mates* | | | *CI* | | | | | *p-value* | | | | |
| Intercept | | 2.12 | | | 2.01 –  2.23 | | | | < 0.001 | | | 1.60 | | | | 1.47 –  1.72 | | | < 0.001 | | | | 2.07 | | | | | 1.96 –  2.19 | | | | < 0.001 | | | | | 3.58 | | | | | 2.23 –4.92 | | | | < 0.001 | | | |
| Tumor group  [ANF] | | -0.47 | | | -0.63 –  -0.30 | | | | < 0.001 | | | -0.51 | | | | -0.70 –  -0.31 | | | < 0.001 | | | | -0.50 | | | | | -0.67 –  0.32 | | | | < 0.001 | | | | | 2.64 | | | | | 0.67 –  4.60 | | | | 0.009 | | | |
| Tumor group  [MPNST] | | -0.76 | | | -0.89 –  -0.63 | | | | < 0.001 | | | -0.82 | | | | -0.97 –  -0.66 | | | < 0.001 | | | | -0.79 | | | | | -0.93 –  -0.65 | | | | < 0.001 | | | | | 7.90 | | | | | 6.38 –  9.43 | | | | < 0.001 | | | |
| Reader  [2] | | 0.01 | | | -0.06 –  0.08 | | | | 0.799 | | | -0.01 | | | | -0.01 –  0.08 | | | 0.862 | | | | 0.00 | | | | | -0.08 –  0.08 | | | | 0.958 | | | | | 0.02 | | | | | -0.82 –  0.85 | | | | 0.972 | | | |
| Random effects | |  | | | |  | | |  | | |  | | |  | | | | |  | | |  | | | | |  | | | | |  | | | |  | | | |  | | | | | |  | | |
| σ^2^ | | | 0.04 | | | |  | | |  | | | 0.07 | | | |  | | | |  | | | | 0.05 | | | |  | | | | |  | | | | | 5.84 | | | | |  | | | | |  |
| τ_00_ | | | 0.05 | | | |  | | |  | | | 0.06 | | | |  | | | |  | | | | 0.05 | | | |  | | | | |  | | | | | 8.09 | | | | |  | | | | |  |
| N | | | 34 | | | |  | | |  | | | 34 | | | |  | | | |  | | | | 34 | | | |  | | | | |  | | | | | 34 | | | | |  | | | | |  |
| Observations | | | | 132 | | | |  | | |  | | | 132 | | | |  | | | |  | | | | 132 | | | |  | | | | |  | | | 132 | | | | |  | | | | |  | |

***Supplementary Tab. 2) Potential SUV_max-_based cut-off values for BPNST, ANF, and MPNST discrimination.***

| **Discrimination** | **Cut-off**  **SUV_max_** | **Sensitivity**  **[%] (95%-CI)** | **Specificity**  **[%] (95%-CI)** | **Positive predictive value [%] (95%-CI)** | **Negative predictive value [%] (95%-CI)** |
| --- | --- | --- | --- | --- | --- |
| 1)  BPNST  vs.  ANF+MPNST | 2.0 | 100 | 37.18 (22.41 – 54.81) | 52.43 (39.05 – 65.47) | 100 |
|  | 3.0 | 96.30 (76.45-99.52) | 56.41 (37.66-73.49) | 60.47 (44.39 – 74.55) | 95.65 (53.99 – 99.76) |
|  | 4.0 | 92.59 (74.62 – 98.15) | 71.80 (55.36 – 83.94) | 69.44 (51.07 – 83.19) | 93.33 (73.83 – 98.58) |
|  | 4.5 | 85.19 (62.71 – 95.16) | 76.92 (62.61 – 86.90) | 71.88 (52.70 – 85.43) | 88.24 (68.51 – 96.28) |
|  | 5.0 | 85.19 (62.71 – 95.16) | 92.31 (77.35 – 97.68) | 88.46 (64.22 – 97.04) | 90.00 (73.45 – 96.70) |
|  | 6.0 | 62.96 (40.68 – 80.82) | 100 | 100 | 79.59 (65.94 – 88.71) |
|  | 7.0 | 48.15 (28.82 – 68.04) | 100 | 100 | 73.59 (60.80 – 83.34) |
| 2)  BPNST+ANF vs.  MPNST | 2.0 | 100 | 29.00 (17.81-43.50) | 31.07 (20.84 – 43.56) | 100 |
|  | 3.0 | 93.75 (62.73 – 99.26) | 44.00 (28.91 – 60.28) | 34.88 (22.51 – 49.70) | 95.65 (53.99 – 99.76) |
|  | 4.0 | 93.75 (62.73 – 99.26) | 58.00 (44.10 – 70.74) | 41.67 (26.69 – 58.35) | 96.67 (73.74 – 99.67) |
|  | 4.5 | 87.50 (57.33 – 97.33) | 64.00 (49.30 – 76.47) | 43.75 (26.59 – 62.55) | 94.12 (77.29 – 98.69) |
|  | 5.0 | 87.50 (57.33 – 97.33) | 76.00 (58.13 – 87.84) | 53.85 (30.98 – 75.20) | 95.00 (83.08 – 98.66) |
|  | 6.0 | 81.25 (51.81 – 94.58) | 92.00 (78.54 – 97.31) | 76.47 (37.61 – 94.60) | 93.88 (85.27 – 97.60) |
|  | 7.0 | 75.00 (47.19 – 90.97) | 98.00 (86.02 – 99.74) | 92.31 (25.36 – 99.77) | 92.45 (84.02 – 96.61) |
